# Prevalence of thyroid carcinoma in nodules with thy 3 cytology: the role of preoperative ultrasonography and strain elastography

**DOI:** 10.1186/s13044-021-00098-x

**Published:** 2021-04-09

**Authors:** Giorgos Pikis, Eleni Kandaraki, Demetris Lamnisos, Sereen Abbara, Katerina Kyriakou, Aliki Economides, Panayiotis A. Economides

**Affiliations:** 1 European University Cyprus, 6 Diogenes Street, Engomi, 2404 Nicosia, Cyprus; 2 Cyprus Institute of Neurology and Genetics, Nicosia, Cyprus; 3 Histopathology & Cytology Medical Center, Nicosia, Cyprus; 4 Thyroid & Endocrinology Center, Engomi, Nicosia, Cyprus

**Keywords:** Thyroid carcinoma, Thy3 cytology, Thyroid ultrasound, Strain elastography

## Abstract

**Background:**

Fine needle aspiration (FNA) cytology, the gold standard in assessing thyroid nodules, is limited by its inability to determine the true risk of malignancy in Thy 3 nodules. Most patients with Thy3 cytology undergo surgery to establish a histologic diagnosis. The aims of this study were to evaluate the prevalence of malignancy in Thy3 nodules, to examine the ultrasound (US) characteristics that are associated with a high cancer risk and to assess the role of real-time strain elastography.

**Methods:**

Retrospective cohort study of 99 nodules with Thy3 cytology in 99 patients who underwent thyroidectomy over a three-year period. Grayscale US, Doppler and real-time strain elastography data were evaluated.

**Results:**

Eighty-one nodules (81.82%) were benign, 18 (18.18%) were malignant, and almost all were papillary thyroid carcinoma (PTC). Univariable analysis revealed irregular margins (*p* = 0.02), ill-defined borders (*p* ≤ 0.001), a taller than wide shape (*p* ≤ 0.001) and the elasticity score (*p* = 0.02) as significant predictors of malignancy. Multivariable analysis showed that ill-defined borders and the elasticity score were significant and independent factors associated with malignancy. All soft nodules (elasticity scores 1–2) were benign (sensitivity 100%, specificity 33%, NPV 100%, and PPV 23%). There was a higher rate of malignancy in Thy3a nodules than in Thy3f nodules (42.86% versus 11.54%) (*p* ≤ 0.001).

**Conclusions:**

Irregular margins, ill-defined borders, a taller than wide shape and low elasticity were associated with malignancy. Elastography should be performed when evaluating Thy3 nodules.

## Introduction

Thyroid nodules are extremely common and are estimated to be found in up to 70% of the population when using high-resolution ultrasonography [1]. Although most nodules are benign, approximately 15% are malignant and can often present with aggressive histopathologic features [2, 3]**.** Fine needle aspiration (FNA), most often performed under US guidance, is currently the most utilized procedure to diagnose malignancy. However, FNA cytology is limited by its inability to differentiate benign follicular adenoma, follicular carcinoma and the follicular variant of papillary carcinoma. This “indeterminate” Thy3 category is subclassified into Thy3a and Thy3f and has a 15–30% risk of malignancy [4–6].

The most challenging aspect in the management of thyroid nodules is the investigation of the Thy3 category [7]. Although ultrasonography is the most effective modality for predicting malignancy, an accurate preoperative differentiation of carcinoma from adenoma is difficult. The ATA guidelines recommend surgery for a histopathologic diagnosis if the cytologically nondiagnostic nodule has a highly suspicious sonographic pattern [8]. However, the accuracy of suspicious thyroid ultrasound features is variable and cannot be used to diagnose malignancy. As the majority of thyroidectomies are performed for benign lesions, an accurate risk stratification will help to reduce unnecessary surgeries and avoid complications.

More recently, molecular markers have been proposed for use to “rule in” or “rule out” the presence of malignancy in thyroid nodules with indeterminate cytology. Some molecular markers offer a high positive predictive value (PPV) in suggesting malignancy [9], while others offer a high negative predictive value (NPV) in suggesting a benign nodule [10]. However, these tests are costly and not readily available in all countries. Furthermore, there is no single optimal molecular test that can definitively diagnose malignancy, and long-term outcome data on the clinical use of these markers are lacking [8, 11].

Real-time elastography (RTE) or “electronic palpation” has also been proposed as a noninvasive tool to modify cancer risk assessment and differentiate benign from malignant nodules [12]. However, as its performance is variable and dependent on the operator, device and technique, the ATA guidelines do not recommend its universal use. The AACE/ACE/AME and ETA guidelines (EU-TIRADS), however, state that elastography may be used as a complimentary tool to grayscale sonography due to its high NPV [13, 14]. In clinical practice, RTE is often performed as an extension of conventional ultrasonography, and it can easily be performed because it is noninvasive and painless. There are two main elastography techniques: strain elastography, which assesses tissue elasticity after external compression causing tissue displacement, and shear wave elastography, which assesses tissue elasticity by measuring propagation of the shear wave [15].

The purpose of this study was to evaluate the prevalence of malignancy in a cohort of Thy3 nodules on the island of Cyprus, examine the ultrasound characteristics that are associated with a high cancer risk and assess the role of real-time strain elastography in evaluating these nodules.

## Materials and methods

This was a retrospective cohort study of 99 nodules with Thy 3 cytology in 99 consecutive patients who underwent thyroidectomy based on cytological results over a three-year period (January 2016–December 2018). Preoperatively, all patients underwent a clinical evaluation and thyroid ultrasound examination on a GE Logiq E9 system with an ML 6–15 linear probe and strain elastography software at the Thyroid & Endocrinology Center in Nicosia, Cyprus, a referral clinic for thyroid diseases. This was followed by US-guided FNA by an endocrinologist experienced in endocrine neck US [16], and the cytology slides were reviewed by a cytologist specializing in thyroid cytology.

The data recorded included sex, age, weight, height and body mass index (BMI). The nodule characteristics recorded included size, solitary or multinodular, parenchymal composition, echogenicity, vascularity, irregularity of margins, calcifications, ill-defined borders and a taller than wide shape. Elasticity was evaluated by comparing the nodule elasticity to that of the normal adjacent thyroid parenchyma (qualitative strain elastography: standard blue-red-green color map). Elastography scores were recorded according to Asteria [17], where an elasticity score of 1–2 indicates an elastic-soft or mostly deformable nodule and a score of 3–4 indicates a less deformable-low elasticity or hard nodule. The diagnosis of autoimmune thyroid disease (Hashimoto’s thyroiditis and Graves' disease) was based on the patient’s history, clinical data, thyroid autoantibodies and ultrasound examination.

The cytological diagnosis was categorized based on the Royal College of Pathologists reporting [18]. The Thy3 category is divided into Thy3f, follicular or Hurthle cell neoplasm; and Thy3a, cytological nuclear or architectural atypia or other features that raise the possibility of neoplasia. After surgical resection, the histopathology results of the studied Thy3 nodules were recorded and compared to the cytological diagnosis. Patients with carcinomas occurring outside the studied Thy3 nodules were not included in the malignant category. The final histology was considered malignant only if cancer was found in the studied Thy3 nodules.

This study was approved by the Cyprus National Bioethics Committee. Patients’ personal information and identities were kept fully confidential throughout the study.

### Statistical analysis

The following factors were examined as possible factors associated with malignancy: sex, age (< 55 vs ≥55 years old), BMI (≤30 vs > 30), Hashimoto’s thyroiditis/Graves’ disease, ultrasound characteristics, elasticity and Thy3f and Thy3a cytology. These factors were analyzed by a series of chi-square tests and univariable logistic regression models that considered each factor separately. Age, BMI and tumor size were also considered continuous variables. Multiple logistic regression was used to simultaneously consider all the statistically significant risk factors. The association of malignancy with each factor is expressed as the OR (odds ratio) and 95% CI (confidence interval). Regarding the elasticity score, all nodules with an elasticity score of 1–2 were considered benign, and for this outcome, the Bayesian logistic regression model was used. This model provides the posterior mean of the odds ratio along with the 95% credible interval. All statistical analyses except for the Bayesian logistic regression model were performed using the statistical package SPSS 20, and statistical significance was set at *p* < 0.05. The Bayesian logistic regression model was performed in R software [19] with the statistical package rstanarm [20].

## Results

There were 79 females (79.79%) and 20 males (20.21%), with a mean age of 49.3 (SD = 13.2) years. Thirty-one (31.31%) patients had autoimmune thyroiditis. Total thyroidectomy was performed in 81 patients, and lobectomy was performed in 18. Histopathological examination of the 99 Thy3 nodules showed that 81 nodules (81.82%) were benign, 18 nodules (18.18%) were malignant, 17 nodules were papillary thyroid carcinoma, and one nodule was Hurthle cell carcinoma. However, micropapillary thyroid carcinoma was found at a different nodule in 8 patients among the 81 in the histologically benign Thy3 nodule group. As the main outcome of interest was the diagnosis of malignancy only in the studied Thy3 nodules, these 8 patients were included in the benign Thy3 nodule group. If the additional microcarcinomas were included, then the cumulative rate of malignancy in our resected thyroid glands became 26/99 (26%).

Patient and disease characteristics are shown in Table 1. There were no significant differences between patients with benign and malignant Thy3 nodules in terms of sex, age, BMI or autoimmune thyroiditis.

**Table 1:** Demographic and clinical characteristics

Characteristics	Overall	Benign Thy3	Malignant Thy3	*P* - value†	Odds ratio (95% confidence interval)
Number of patients	99 (100)	81 (81.81)	18 (18.18)		
Sex					
female	79 (79.79)	64 (81.01)	15 (18.99)	0.68	1.33 (0.34, 5.12)
male	20 (20.21)	17 (85)	3 (15)		Ref
Age (years)					
< 55	64 (64.64)	52 (81.25)	12 (18.75)	0.84	1.12 (0.38, 3.29)
≥ 55	35 (35.35)	29 (82.85)	6 (17.14)		Ref
Age (years)	Mean: 49.3	Mean: 49.5	Mean: 48.4	0.75^¶^	1.01 (0.97, 1.05)
	SD: 13.2	SD: 13.1	SD: 13.7		
BMI (kg/m^2^)					
≤ 30	78 (78.78)	64 (82.05)	14 (17.95)	0.91	Ref
> 30	21 (21.21)	17 (80.95)	4 (19.04)		1.08 (0.31, 3.69)
BMI (kg/m^2^)	Median: 25.6	Median: 25.2	Median: 26.45	0.76^¶^	0.99 (0.89, 1.08)
	IQR: 6.3	IQR: 6.8	IQR: 6.3		
Hashimoto’s /Graves’					
Yes	31 (31.31)	25 (80.6)	6 (19.4)	0.84	1.12 (0.38, 3.32)
No	68 (68.68)	56 (82.4)	12 (17.6)		Ref

Variables are n (%) unless stated otherwise. †*p*-value from the χ2 test or Fisher’s exact test.

¶*p*-value from the independent samples t-test or the Mann-Whitney test.

The thyroid ultrasound characteristics of the examined nodules are shown in Table 2. There was a statistically significant difference in the rate of irregular margins (*p* = 0.02), ill-defined borders (*p* ≤ 0.001) and being taller than wide (*p* ≤ 0.001) between benign and malignant nodules. No statistically significant difference was found between the groups in terms of size, solitary/multinodular status, echogenicity, vascularity or the presence of calcifications. Multivariable analysis further showed that ill-defined borders (OR 27.88, 95% CI 4.59–169.37) was a significant and independent factor associated with malignancy.

**Table 2:** Ultrasound characteristics of Thy3 nodules

Characteristics	Overall	Benign Thy3	Malignant Thy3	*P* - value†	Odds ratio (95% confidence interval)	Adjusted odds ratio^a^ (95% confidence interval)	*p*-value‡
Number of nodules	99 (100)	81 (81.81)	18 (18.18)				
Size							
≤ 10 mm	61 (61.6)	50 (82.0)	11 (18.0)	0.96	Ref	_	
> 10 mm	38 (38.4)	31 (81.6)	7 (18.4)		1.03 (0.36, 2.93)		
Size (mm)	Median: 8.3	Median: 8.6	Median: 7.5	0.51^¶^	1.03 (0.94, 1.13)	_	
	IQR: 7.4	IQR: 7.2	IQR: 7.7				
Solitary	6 (6.06)	5 (83.33)	1 (16.66)	0.92	Ref	_	
Multinodular	93 (93.93)	76 (81.72)	17 (18.28)		1.12 (0.12, 10.2)		
Nodule characteristics							
Solid hypoechoic	81 (81.81)	64 (79.01)	17 (20.99)	0.18	4.52 (0.56, 36.38)	–	
Solid isoechoic	18 (18.18)	17 (94.44)	1 (5.55)		Ref		
Vascularity (color Doppler)							
Non vascular (no flow)	63 (63.63)	49 (77.8)	14 (22.2)	0.17	2.29 (0.69, 7.57)	–	
Increased vascularity (peripheral and/or central flow)	36 (36.36)	33 (88.9)	3 (11.1)		Ref		
Margins							
Regular	85 (85.85)	72 (84.70)	13 (15.30)	0.02	4.56 (1.34, 15.48)	1.21 (0.16, 8.87)	0.86
Irregular	14 (14.14)	9 (64.28)	5 (35.71)		Ref	Ref	
Calcifications							
Yes	19 (19.19)	14 (73.68)	5 (26.31)	0.33	1.84 (0.57, 6.00)	–	
No	80 (80.80)	67 (83.75)	13 (16.25)		Ref		
Ill-defined borders							
Yes	14 (14.14)	3 (21.42)	11 (78.58)	≤0.001	40.9 (9.2, 182.7)	27.88 (4.59, 169.37)	0.00
No	85 (85.85)	78 (91.76)	7 (8.24)		Ref	Ref	
Taller than wide shape							
Yes	14 (14.14)	6 (42.85)	8 (57.15)	≤0.001	10.0 (2.87, 34.81)	4.80 (0.88, 26.19)	0.07
No	85 (85.85)	75 (88.23)	10 (11.76)		Ref	Ref	

Variables are n (%) unless stated otherwise. †*p*-value from the χ2 test or Fisher’s exact test.

¶*p*-value from the Mann-Whitney test.

^a^Adjusted for margins, ill-defined borders, taller than wide shape, elastography and Thy3.

‡*p*-value from multiple logistic regression.

Seventy-nine nodules (79.69%) underwent elastography (Table 3). There was a statistically significant difference in the rate of low elasticity when comparing benign and malignant nodules (*p* = 0.02). All 22 nodules (100%) with an elasticity score of 1–2 (soft) were benign (sensitivity 100%, specificity 33%, NPV 100%, and PPV 25%). Multivariable analysis showed that elasticity was a significant and independent factor associated with malignancy (OR 19.85, 95% CrI 1.56–637.13).

**Table 3:** Elastography findings

Characteristics	Overall	Benign Thy3	Malignant Thy3	*P* - value†	Odds ratio (95% credible Interval)	Adjusted odds ratio^a^ (95% credible interval)
Scores 1 & 2 (Soft)	22 (27.84)	22 (100)	0 (0)	0.02	Ref	Ref
Scores 3 & 4 (Hard)	57 (72.15)	44 (77.19)	13 (22.80)		20.77 (2.22, 660.49)	19.85 (1.56, 637.13)

Variables are n (%) unless stated otherwise. †*p*-value from Fisher’s exact test.

^a^Adjusted for margins, ill-defined borders, taller than wide shape and Thy3.

Seventy-eight (78.78%) nodules had Thy3f cytology, and 21 (21.21%) nodules had Thy3a cytology (Table 4). Sixty-nine (88.46%) Thy3f nodules were histologically benign, and 9 (11.54%) were malignant. Twelve (57.14%) Thy3a nodules were histologically benign, and 9 (42.86%) were malignant (*p* ≤ 0.001).

**Table 4:** Thy3f and Thy3a cytology

Cytology	Overall	Benign Thy3	Malignant Thy3	*P* - value†	Odds ratio (95% confidence interval)	Adjusted odds ratio^a^ (95% confidence interval)	*p*-value‡
Thy3f	78 (78.78)	69 (88.46)	9 (11.54)	≤0.001	Ref	Ref	0.77
Thy3a	21 (21.21)	12 (57.14)	9 (42.86)		5.75 (1.90, 17.43)	1.12 (0.53, 2.38)	

Variables are n (%) unless stated otherwise. †*p*-value from the χ2 test.

^a^Adjusted for margins, ill-defined borders, taller than wide shape, elastography and Thy3.

‡*p*-value from multiple logistic regression.

Table 5 shows the histopathologic diagnosis according to the cytological subcategory. Fifty-three (67.94%) Thy3f nodules were microfollicular adenomas, and 16 (20.51%) were Hurthle cell adenomas. Three (3.84%) nodules were classic PTC, 5 (6.41%) were follicular variant PTC, and 1 (1.28%) was Hurthle cell carcinoma. Eight (38.09%) Thy3a nodules were microfollicular adenomas, 4 (19.04%) were Hurthle cell adenomas, and 9 (42.86%) were classic PTCs.

**Table 5:** Cytology with the corresponding histological diagnosis

Cytology	Overall	Benign Thy3	Malignant Thy3	Microfollicular adenoma	Hurthle cell adenoma	PTC classic	PTC follicular variant	Hurthle cell ca
Thy3f	78 (100)	69 (88.46)	9 (11.54)	53 (67.94)	16 (20.51)	3 (3.84)	5 (6.41)	1 (1.28)
Thy3a	21 (100)	12 (57.14)	9 (42.86)	8 (38.09)	4 (19.04)	9 (42.86)	0 (0)	0 (0)

Variables are n (%) unless stated otherwise

## Discussion

The management of nodules with Thy3 or “indeterminate” cytology, which is reported in up to 30% of FNAs, is challenging, as no single diagnostic test can offer accurate results. Unfortunately, the majority of these patients undergo surgery to establish a histologic diagnosis. Our study aimed to examine the prevalence of malignancy in nodules with Thy3 cytology in a cohort of patients in Cyprus and to determine whether there are US or elastography features that are associated with malignancy.

We found an 18.18% prevalence of malignancy in our Thy3 nodules, which is higher than that reported by Kaliszewski et al. [21], who showed a prevalence of 10.2%, and lower than that reported in other similar studies, with malignancy rates ranging from 27 to 45.1% [22, 23]**.** When we examined the Thy3 subcategories, we observed that the prevalence of malignancy in nodules with Thy3f cytology was 11.54%, whereas a much higher prevalence of 42.86% was observed in nodules with Thy3a cytology. Alexander et al. showed that both Thy3a and Thy3f had similar rates of malignancy (30.4, 29.2%) [24], whereas Brophy et al. showed that the incidence of malignancy was higher in Thy3f than in Thy3a, but this was not statistically significant [25]. In disagreement with our findings, Mullen et al. [26] showed a malignancy rate of only 7% in their Thy3a cytology cases. These discrepancies question the usefulness of the subcategorization of the Thy 3 category to Thy3a and Thy3f [24].

Our results showed that irregular margins, ill-defined borders and a taller than wide shape were associated with malignancy. Deng et al. showed that ill-defined margins was a risk factor for intermediate-risk nodules and that the probability of malignancy increased as the number of risk factors increased [27]. Ocal et al. showed that irregular margins was an independent risk factor for malignancy on US, and they suggested a nomogram to predict malignancy in indeterminate nodules by using clinical, cytological and ultrasound features [28]. In a meta-analysis by Gao et al. [29], it was shown that US was helpful in differentiating benign and malignant Bethesda class III nodules and that nodules with more suspicious features were more likely to be malignant. Gomes-Lima et al. proposed that molecular testing could be avoided in indeterminate nodules when they were ultrasonographically high risk, as the specificity of cancer diagnosis is non-inferior to molecular testing [30]**.** Hypoechogenicity is a significant predictor of malignancy in Bethesda class III nodules [21]. In our study, although hypoechogenicity was associated with an increased risk of malignancy, this was not statistically significant.

Real-time elastography (RTE) was performed in most of our Thy3 nodules, despite the debate regarding its usefulness. Our results showed that none of the high elasticity (soft) nodules were malignant, and our high sensitivity and NPV is in agreement with the study by Chiorean et al., who also reported high sensitivity [31]**.** Stoian et al. showed that combining elastography and conventional ultrasound increased the diagnostic accuracy in nodules with indeterminate cytology, especially in those with low stiffness [32]. Similarly, Friedrich-Rust et al. showed that strain elastography could be used as an additional US tool that improves the diagnostic value of US for the exclusion of malignant nodules [33]. Shear Wave Elastography (SWE) has been compared to molecular testing in nodules with indeterminate cytology, and it was shown that both modalities demonstrate similar diagnostic performance and are independent predictors of malignancy [34]. Although there is currently no consensus as to which elastography technique is better, the operator dependent strain elastography was shown to perform better than SWE in distinguishing malignancy in indeterminate cytology thyroid nodules [35]. The European Federation of Societies for Ultrasound in Medicine and Biology (EFSUMB) recommends elastography as an additional tool for thyroid lesion differentiation that may be also used to guide follow up of the lesions negative for malignancy at FNA [36].

Our study has several limitations. First, this was a retrospective study performed at a single center; thus, our results may not represent the whole population of Cyprus. However, to the best of our knowledge, this is the first study in Cyprus to examine the role of US characteristics and elastography in evaluating Thy3 nodules. Second, our study included only patients who underwent surgery, so there may be potential selection bias. However, as histopathology is the only method to establish the presence of malignancy in Thy3 nodules, this bias cannot be avoided. Third, as in our study almost all malignant cases were papillary thyroid carcinoma, our findings cannot represent follicular or Hurthle cell carcinoma or other tumors with a follicular architecture. Fourth, as the elastography technique was performed by a single examiner, the results cannot be generalized, as this technique is dependent on the operator and machine. Further larger prospective studies should be undertaken to evaluate the role of strain elastography in this regard.

## Conclusion

In summary, the prevalence of malignancy in our cohort of Thy3 nodules was 18.18%, with a higher rate of malignancy in Thy3a nodules than in Thy3f nodules. Almost all of our malignant Thy3 nodules were papillary thyroid carcinoma, and most of our benign Thy3 nodules were microfollicular adenomas. Irregular margins, ill-defined borders, a taller than wide shape and low elasticity were associated with malignancy. Real-time strain elastography should be performed when evaluating Thy3 “indeterminate” nodules.

## Data Availability

The data used to support the findings of this study are included within the article.

## Abbreviations

ΑΤΑ: American Thyroid Association
BMI: Body Mass Index
CI: Confidence Interval
CrI: Credible Interval
EFSUMB: The European Federation of Societies for Ultrasound in Medicine and Biology
ETA: European Thyroid Association
FNA: Fine Needle Aspiration
NPV: Negative Predictive Value
OR: Odds Ratio
PPV: Positive Predictive Value
PTC: Papillary Thyroid Carcinoma
RTE: Real Time Elastography
SWE: Shear Wave Elastography
US: Ultrasound
